# IGF-1R inhibition sensitizes breast cancer cells to ATM-related kinase (ATR) inhibitor and cisplatin

**DOI:** 10.18632/oncotarget.10862

**Published:** 2016-07-27

**Authors:** Ciara H. O'Flanagan, Sandra O'shea, Amy Lyons, Fionola M. Fogarty, Nuala McCabe, Richard D. Kennedy, Rosemary O'Connor

**Affiliations:** ^1^ School of Biochemistry and Cell Biology, University College Cork, Cork, Ireland; ^2^ Almac Diagnostics, Craigavon, Northern Ireland, UK; ^3^ Centre for Cancer Research and Cell Biology, Queen's University Belfast, Belfast, Northern Ireland, UK

**Keywords:** IGF-1R, DNA damage, breast cancer, TKI

## Abstract

The complexity of the IGF-1 signalling axis is clearly a roadblock in targeting this receptor in cancer therapy. Here, we sought to identify mediators of resistance, and potential co-targets for IGF-1R inhibition. By using an siRNA functional screen with the IGF-1R tyrosine kinase inhibitor (TKI) BMS-754807 in MCF-7 cells we identified several genes encoding components of the DNA damage response (DDR) pathways as mediators of resistance to IGF-1R kinase inhibition. These included ATM and Ataxia Telangiectasia and RAD3-related kinase (ATR). We also observed a clear induction of DDR in cells that were exposed to IGF-1R TKIs (BMS-754807 and OSI-906) as indicated by accumulation of γ-H2AX, and phosphorylated Chk1. Combination of the IGF-1R/IR TKIs with an ATR kinase inhibitor VE-821 resulted in additive to synergistic cytotoxicity compared to either drug alone. In MCF-7 cells with stably acquired resistance to the IGF-1R TKI (MCF-7-R), DNA damage was also observed, and again, dual inhibition of the ATR kinase and IGF-1R/IR kinase resulted in synergistic cytotoxicity. Interestingly, dual inhibition of ATR and IGF-1R was more effective in MCF-7-R cells than parental cells. IGF-1R TKIs also potentiated the effects of cisplatin in a panel of breast cancer cell lines. Overall, our findings identify induction of DDR by IGF-1R kinase inhibition as a rationale for co-targeting the IGF-1R with ATR kinase inhibitors or cisplatin, particularly in cells with acquired resistance to TKIs.

## INTRODUCTION

The Insulin/Insulin-like growth factor-1 receptor (IR/IGF-1R) system is widely expressed, and while Insulin signalling is central to glucose metabolism, IGF-1 signalling has an essential role in regulating multiple cellular pathways that govern cell fate, cell proliferation, growth, and cancer progression [1–3]. The canonical IGF signalling pathway recruited by the IRS scaffolding proteins activates PI3-Kinase, Akt, and Ras, all of which are potent oncogenes that are deregulated in many cancers [4, 5]. The IGF-1R can also be recruited to sites of focal adhesions to activate the FAK and MAP kinase pathways and promote cell motility and invasiveness [6–11]. Such dynamic cooperative signalling between IGF-1R and Integrins may contribute to invasiveness and metastasis of cancer cells (reviewed in [12]). IGF-1R and Integrin cooperation has also been linked to epithelial-to-mesenchymal transition (EMT), a key step in metastasis [13] and to resistance to targeted therapies [14].

Several IGF-1R/IR inhibitors have been tested in clinical trials, but these inhibitors have shown limited efficacy [15, 16]. Despite these poor results, individual responses of patients within these trials were often striking. Figitumumab treatment led to a complete resolution of pulmonary metastasis [17] and Ganitumab treatment led to a 28 month sustained durable complete response in a Ewing's sarcoma patient [18]. However, many other Phase II/III clinical trials, in particular breast, lung and pancreatic cancers, have failed to show clinical benefit in unselected patients. This is likely due to the complexity of the IR/IGF-1R signalling axis, compensatory mechanisms through other receptors, and a lack of predictive tumour- and patient-selective biomarkers of response [15, 16, 19]. Much effort is now focused on understanding the cellular effects of IGF-1R inhibition, the mechanisms of resistance and sensitivity, the identification of reliable predictive biomarkers of response, and testing options for combination with other therapies [20]. Resistance to IGF-1R inhibition can be mediated by compensatory up-regulation of the IR [21, 22] or Her2 Receptors [23] signalling pathways activated by MEK [24]; or mTOR activity. Compensatory signalling through IGF-1R can also mediate resistance to EGFR TK inhibitors [25]. However, effective combination therapy of IGF-1R inhibitors with chemotherapy or TK inhibitors has not yet been demonstrated.

The possibility of combining IGF-1R inhibition with induction of DNA damage by radiotherapy or cisplatin-based compounds is also being explored. Double or single-stranded breaks in DNA normally activate DNA damage repair mechanisms in order to maintain genome stability. These involve activation of the ataxia telangiectasia mutated (ATM) and ataxia telangiectasia and Rad3-related (ATR) kinases, which lead to cell cycle arrest and DNA repair. Suppression of IGF-1R signalling has been shown to enhance radiosensitivity and impair activation of ATM [26], and to sensitize ovarian and colon cancer cells to cisplatin [27, 28]. IGF-1R inhibition also enhances radio-sensitivity in prostate cell lines due to impaired double-stranded DNA repair mechanisms [29]. Inhibition of ATR has also been suggested as an attractive approach for sensitization of tumour cells to DNA damaging agents. However the impact of dual inhibition of ATR with RTKs such as the IGF-1R has not yet been investigated.

The aim of this study was to identify mediators of sensitivity or resistance to IGF-1R kinase inhibition. Using a targeted RNAi screen we identified a number of genes that sensitize cells to IGF-1R inhibition. Among these the DNA damage and repair genes were strongly represented suggesting they may be suitable targets for co-inhibition with IGF-1R inhibition.

## RESULTS

### SiRNA screen identifies potential biomarkers and targets for IGF-1R inhibition

**Figure 1:**
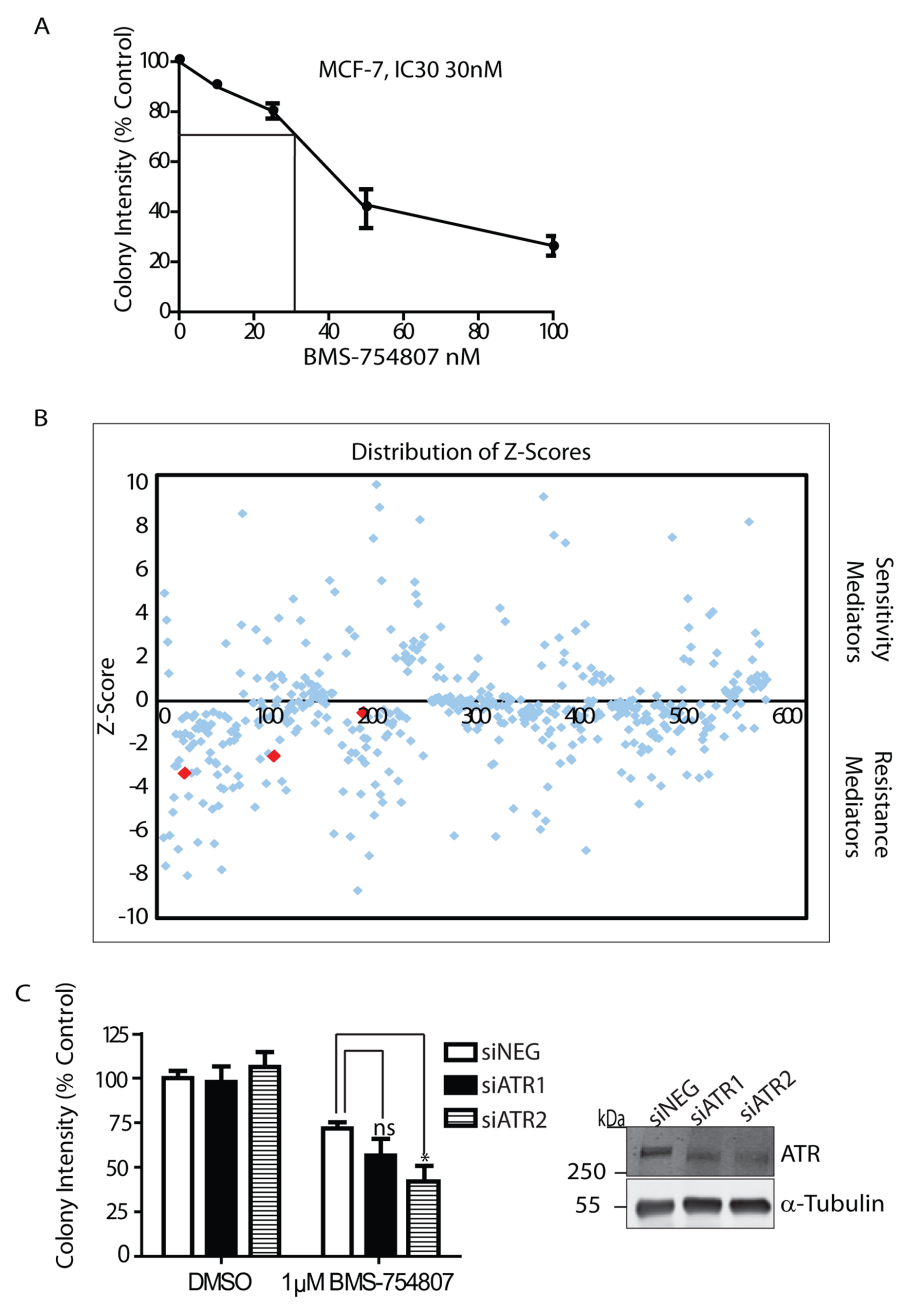
Tumour suppressor and oncogene siRNA screen to identify potential biomarkers and targets for IGF-1R inhibition **A.** Survival curve for MCF-7 cells incubated with 30nM BMS-754807 for determination of IC30. Cells were seeded into colony formation assays and allowed to grow for 14 days. Values are expressed as colony intensity as a percentage of the untreated control. **B.** Distribution of Z-Scores generated from the tumour suppressor and oncogene siRNA screen. Positive scores indicate potential mediators of sensitivity to BMS-754807, while negative scores indicate potential mediators of resistance to the drug. Red points show Z-scores for ATR. Graph is representative of two independent screens, and those Z-Scores which were robust across both screens were carried forward as potential targets. **C.** Survival graph showing knockdown of ATR sensitizes ZR-75-1 cells to BMS-754807. Values are expressed as colony intensity as a percentage of the untreated control. Exposure of control cells to 1μM of BMS-754807 reduced colony formation to 75% of the untreated control. Suppression of ATR with two independent siRNA oligonucleotides produced a greater reduction in colony formation (65% and 45%); n = 3 *P<0.05, ns=not significant, Student-T-Test. Suppression of ATR was confirmed by Western blot analysis.

To identify potential mediators of sensitivity or resistance to IGF-1R inhibition, we carried out a functional screen with a customised siRNA library that was selected to target 178 oncogenes or tumour suppressors (Figure 1). BMS-754807 is a reversible ATP competitive dual IGF-1R/IR inhibitor, with an IC_50_ ranging 100nM to 25μM in breast cancer cell lines [30] and a recommended dose of 100mg daily in patients [31]. MCF-7 cells were first incubated with increasing concentrations of the IGF-1R/IR kinase inhibitor BMS-754807 in colony formation assays in order to determine a sub-lethal dose of inhibitor in these cells, which was established at 30nM (Figure 1A). BMS-754807 was found to inhibit phosphorylation of the IGF-1R, and activation of the PI3-K and MAPK pathways in response to IGF-1 (Supplementary Figure S1). Following transfection with the siRNA library in 96 well plates, cells were cultured for 24 hours and then incubated with vehicle or BMS-754807 (30nM) for a further 48 hrs before cell viability was assessed. The results were median-centred, log transformed and normalized to scrambled siRNA. Hits that affected cell viability with robust Z-scores of at least +/− 1 mean absolute deviation (MAD) from the scrambled siRNA were selected and carried forward for pathway analysis, while hits with at least +/− 3MAD were carried forward for validation studies (Figure 1B). Pathway analysis of the hits using DAVID functional annotation tool (http://david.abcc.ncifcrf.gov/content.jsp?file=citation.html) indicated that inhibition of cell cycle arrest and DNA damage and repair pathways conferred sensitivity and in some cases resistance to IGF-1R inhibition (Table 1). Included in these hits was ATR (a key protein involved in DNA damage sensing and response) that when suppressed robustly sensitized MCF-7 cells to BMS-754807 cytotoxicity. The effects of suppressing ATR with siRNA were further tested in the ZR-75-1 ductal breast carcinoma cell line, which was previously shown to be resistant to IGF-1R inhibition (IC_50_ 2μM for BMS-754807) [32]. Using two siRNAs to suppress ATR expression in these cells we observed that ATR knockdown significantly reduced colony formation in the presence of BMS-754807 (Figure 1C). Exposure of control cells to1μM of BMS-754807 reduced colony formation to 75% of the untreated control, while suppression of ATR produced a greater reduction in colony formation (65% and 45%); indicating ATR contributes to resistance to IGF-1R inhibition. These results confirm the effects observed in the siRNA screen and overall indicate that the DNA damage and repair response is engaged in responses to IGF-1R inhibition.

**Table 1:** List of genes identified in siRNA screen that are associated with DNA damage response and confer sensitivity to BMS-754807

Gene	Function	Sensitivity/Resistance to BMS-754807
**ATM**	DNA Damage Sensing	Resistance
**ATR**	DNA Damage Sensing	Resistance
**BCL2**	Anti-apoptosis	Sensitivity
**BLM**	DNA Damage Checkpoint	Resistance
**BRCA1**	DNA Damage Repair	Resistance
**BRCA2**	DNA Damage Repair	Resistance
**CAV1**	DNA Damage Repair	Resistance
**CDKN1A**	Cell Cycle Arrest	Resistance
**CDKN1B**	Cell Cycle Arrest	Sensitivity
**CDKN2B**	Cell Cycle Arrest	Resistance
**CHEK1**	DNA Damage Response/Cell Cycle Arrest	Resistance
**CHEK2**	DNA Damage Response/Cell Cycle Arrest	Sensitivity
**EGR1**	DNA Damage Response	Resistance
**ERCC1**	DNA Damage Repair	Resistance
**FANCD2**	DNA Damage Repair	Resistance
**FANCG**	DNA Damage Repair	Resistance
**FLI1**	DNA Damage Response	Resistance
**GADD45A**	DNA Damage Response/Cell Cycle Arrest	Sensitivity
**HIF1A**	DNA Damage Response	Resistance
**MGMT**	DNA Damage Repair	Sensitivity
**MLH1**	DNA Mismatch Repair	Resistance
**MSH6**	DNA Mismatch Repair	Resistance
**PTCH1**	DNA Damage Protection	Resistance
**RAD51**	DNA Damage Repair	Resistance
**TP53BP2**	Cell Cycle Arrest	Resistance
**TP53I11**	Cell Cycle Arrest	Resistance
**XRCC5**	DNA Damage Repair	Sensitivity
**XRCC6**	DNA Damage Repair	Resistance

### IGF-1R inhibition induces DNA damage and γ-H2AX accumulation

**Figure 2:**
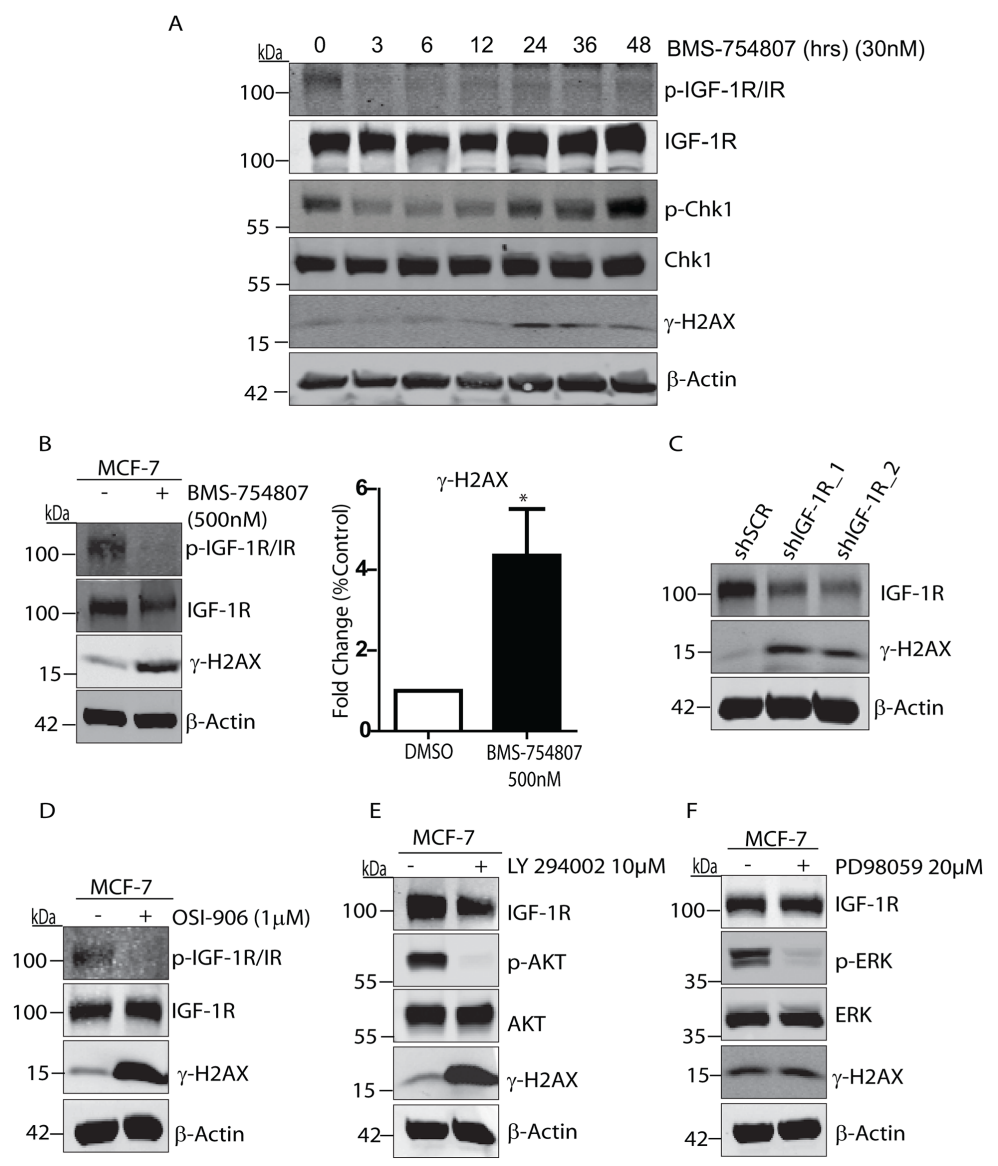
IGF-1R inhibition causes DNA damage and induction of γH2AX **A.** Representative western blot showing BMS-754807 causes DNA damage and activation of ATR, as measured by the induction of γH2AX and phosphorylated Chk1. MCF-7 cells cultured in complete medium were exposed to 30nM BMS-754807 over a time course of 0 to 48 hours, followed by cell lysis and immunoblotting with anti-phospho-IGF-1R/IR, anti-IGF-1R, anti-phospho-Chk1, anti-Chk1, anti-phospho-H2AX (γH2AX) and anti-β-Actin antibodies **B.** MCF-7 cells were exposed to 500nM of BMS-754807 for 24 hours followed by cell lysis and immunoblotting with anti-phospho-IGF-1R/IR, anti-IGF-1R, anti-phospho-H2AX (γH2AX), or anti-β-Actin antibodies. Quantification is from 3 distinct experiments *P<0.01 Student-T-Test. **C.** Whole cell lysates from MCF-7 cells stably expressing pGIPZ Scramble shRNA, pGIPZ shIGF-1R-1, or pGIPZ shIGF-1R-2 vectors were resolved by SDS-PAGE, followed by immunoblotting with anti-IGF-1R, anti-phospho-H2AX (γH2AX), and anti-β-Actin antibodies. **D.** MCF-7 cells were exposed to 1μM of OSI-906 for 24 hours followed by cell lysis and immunoblotting with anti-phospho-IGF-1R/IR, anti-IGF-1R, anti-phospho-H2AX (γH2AX), or anti-β-Actin antibodies. **E.** MCF-7 cells were exposed to 10μM LY294002 (PI3-K inhibitor) for 24 hours followed by cell lysis and immunoblotting with anti-IGF-1R, anti-phospho-AKT, anti-AKT, anti-phospho-H2AX (γH2AX), or anti-β-Actin antibodies. **F.** MCF-7 cells were exposed to 20μM of PD98059 for 24 hours followed by cell lysis and immunoblotting with anti-IGF-1R, anti-phospho-ERK, anti-ERK, anti-phospho-H2AX (γH2AX), or anti-β-Actin antibodies.

We were next interested to investigate the effects of IGF-1R inhibition on the DNA damage response in breast cancer cells. IGF-1R has previously been shown to protect cells from DNA damaging agents [33, 34], and to be critical for DNA repair by homologous recombination following radiation in prostate cancer cells [29]. We first observed that BMS-754807 caused a significant increase in DNA damage along with activation of the ATR pathway, as measured by the formation of γ-Histone2AX (γH2AX) and phosphorylation of Chk1 on Serine 345 respectively (Figure 2A). BMS-754807-induced DNA damage was further verified by immunofluorescence labelling of distinct γH2AX-containing foci that were visible in the nuclei of MCF-7 cells exposed to BMS-754807 (Supplementary Figure S2). This induction of γH2AX in MCF-7 cells is consistent with γH2AX induction by IGF-1R inhibition and IGF-1R depletion observed in prostate cancer cells [35]. Higher concentrations of BMS-754807 also induced DNA damage (Figure 2B) leading to a four-fold increase in γH2AX levels. Induction of γH2AX was significantly reduced upon siRNA-mediated suppression of IGF-1R expression (Supplementary Figure S3) and stable suppression of IGF-1R using shRNA also induced γH2AX (Figure 2C). Thus, inhibition or suppression of IGF-1R is sufficient to induce DNA damage. Moreover, a second TKI targeting the IGF-1R, OSI-906, which strongly suppresses IGF-1 signalling in MCF-7 cells (Supplementary Figure S4) was also shown to increase induction of γH2AX (Figure 2D). Inhibition of the PI3-K pathway, but not the MAPK pathway led to a marked increase in γH2AX induction (Figure 2E and 2F). It is therefore likely that the increase in DNA damage induced by inhibition of the IGF-1R is a result of decreased activity of the PI-3K pathway and not the MAPK pathway. Overall, we conclude that suppression of IGF-1R kinase activity or IGF-1R expression levels is sufficient to induce the DNA damage and DNA repair response pathways in breast cancer cells.

### Combination of ATR and IGF-1R inhibitor decreases clonogenic growth potential

**Figure 3:**
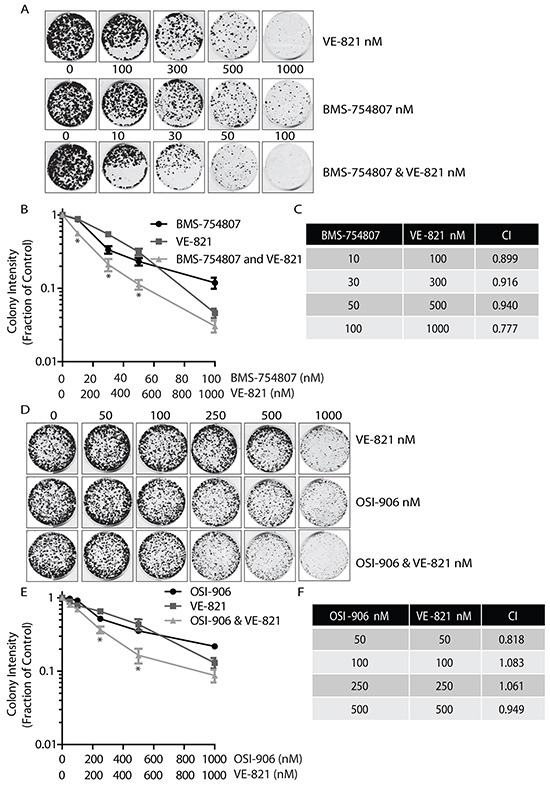
Dual combination of ATR and IGF-1R inhibitors results in decreased colony growth **A.** Dual inhibition of ATR and IGF-1R using VE-821 and BMS-754807 visibly reduces colony numbers. MCF-7 cells were seeded into colony formation assays in complete medium containing increasing concentrations of BMS-754807, VE-821, or BMS-754807 and VE-821 together, and allowed to grow for 14 days. Drugs were replenished every 4 days. The Odyssey Infrared Imaging system was used to produce images of colonies. **B.** Survival curve for MCF-7 cells exposed to increasing concentrations of BMS-754807, VE-821, or BMS-754807 and VE-821 together, expressed as a fraction of the untreated control (Log_10_ scale). Statistical significance was calculated using 2way-Anova, * indicates a significant difference between both BMS-754807 versus BMS-754807/VE-821 combination, and VE-821 versus BMS-754807/VE-821 combination. N=3, P<0.05 **C.** Calculation of Synergy affects using CompuSyn Software. Where a Combination Index (CI) <1, two drugs are synergistic, where CI = 1, two drugs are additive and where a CI>1 two drugs are said to antagonistic. **D.** MCF-7 cells were seeded into colony formation assays and exposed to increasing concentrations of OSI-906, VE-821, or OSI-906 and VE-821 together, and allowed to grow for 14 days. Drugs were replenished every 4 days. The Odyssey Infrared Imaging system was used to produce images of assays. **E.** Survival curve for MCF-7 cells in complete medium containing increasing concentrations of OSI-906, VE-821, or OSI-906 and VE-821 together, expressed as a fraction of the untreated control (Log_10_ scale). Statistical significance was calculated using 2way-Anova, * indicates significant difference between both OSI-906 versus OSI-906/VE-821 combination, and VE-821 versus OSI-906/VE-821 combination. N=3, P<0.05 **F.** Calculation of Synergy affects using CompuSyn Software.

Since inhibition of the IGF-1R promoted a pronounced accumulation of γ-H2AX (Figure 2) and suppression of ATR sensitised cells to IGF-1R inhibition (Figure 1), we next investigated whether dual inhibition of IGF-1R and ATR would be effective. MCF-7 cells were exposed to a range of concentrations of IGF-1R TKI, to the selective ATR inhibitor VE-821 [36] or to a combination of both agents. The effectiveness of VE-821 in preventing ATR activation was confirmed by pre-incubation of cells with VE-821 prior to UV exposure (Supplementary Figure S5). Colonies were then allowed to form for 10-14 days. Combining BMS-754807 and VE-821 led to a visible reduction in colony formation with concentrations of 10nM BMS-754807 and 100nM VE-821 (Figure 3A). This inhibition of colony growth was notable across the range of combinations with total loss of colony formation at a concentration of 100nM BMS-754807 combined with 1000nM of VE-821 (Figure 3B). Synergy analyses with the CompuSyn software [37, 38] indicated that the Combination Index (CI) for BMS-754807 and VE-821 was just below 1. This suggests that the relationship between the two drugs was additive-to-synergistic between concentrations of 10 to 100nM BMS, and 100 to 1000nM VE-821 (Figure 3C).

Since the IGF-1R inhibitor OSI-906 also promoted accumulation of γ-H2AX, we tested dual inhibition with OSI-906 and VE-821 over a range of concentrations. OSI-906 has been tested in clinical trials using 600mg intermittent dosing, leading to a corresponding plasma concentration of 1μM [31]. This also resulted in a more pronounced reduction in colony formation compared to cultures exposed to either agent alone (Figure 3D). OSI-906 and VE-821 in combination consistently decreased colony growth over the entire range of drug concentrations (Figure 3E). Synergy calculations indicated that the relationship between the two drugs was additive for most concentrations, with only 50nM and 500nM concentrations of each drug producing a synergistic relationship (Figure 3F). The dual effect of OSI-906 and VE-821 seemed less effective than the dual effect of BMS-754807 and VE-821 together. This is consistent with the observation that higher concentrations of OSI-906 are required to totally inactivate the kinase activity of the IGF-1R compared with BMS-754807 (Supplementary Figures S1 and S4). Overall the data indicates that the combined use of IGF-1R and ATR inhibitors together is effective in reducing colony growth with a relationship ranging from additive to synergistic.

### MCF-7 cells resistant to BMS-754807 accumulate γH2AX and are sensitive to dual kinase inhibition of IGF-1R and ATR

**Figure 4:**
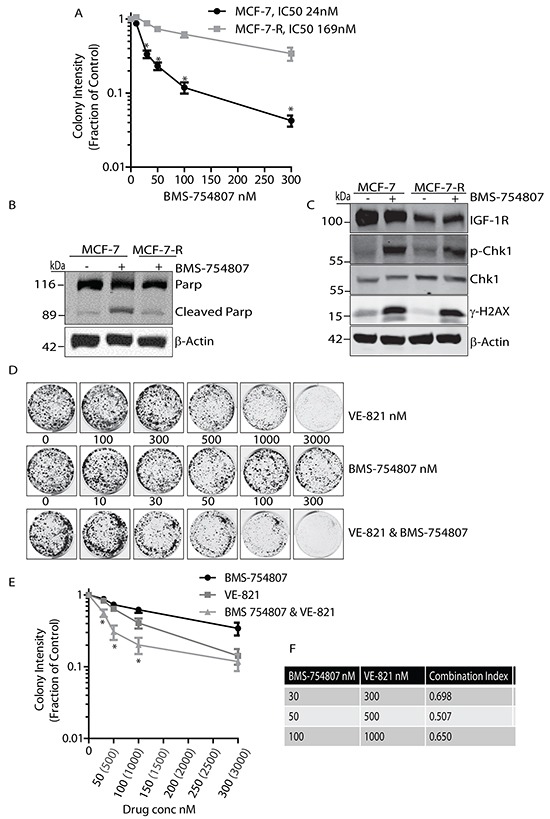
BMS-754807 and VE-821 synergise to reduce colony formation in BMS-754807 Resistant MCF-7 cell line **A.** Survival curve of MCF-7 parental and MCF-7 BMS-Resistant cells treated with increasing concentrations of BMS-754807. Statistical significance was calculated using Student-T-Test, n=3 *P<0.05. **B.** MCF-7 parental and MCF-7-R cells were exposed to 500nM of BMS-754807 for 24 hours followed by cell lysis and immunoblotting with anti-Parp and anti-β-Actin antibodies. **C.** MCF-7 parental and MCF-7-R cells were maintained in complete medium or medium containing 500nM of BMS-754807 for 24 hours followed by cell lysis and immunoblotting with anti-IGF-1R, anti-phospho-Chk1, anti-Chk1, anti-phospho-H2AX (γH2AX), or anti-β-Actin antibodies. **D.** Dual inhibition of ATR and IGF-1R visibly reduces colony numbers. MCF-7-R cells were seeded into colony formation assays and incubated with increasing concentrations of BMS-754807, VE-821, or BMS-754807 and VE-821 together, and allowed to grow for 14 days. Drugs were replenished every 4 days. Odyssey Infrared Imaging system was used to produce images of assays. **E.** Survival curve for MCF-7-R cells incubated with increasing concentrations of BMS-754807, VE-821, or BMS-754807 and VE-821 together, expressed as a fraction of the untreated control (Log_10_ scale). Statistical significance was calculated using 2way-Anova, * indicates significant difference between both BMS-754807 versus BMS-754807/VE-821 combination, and VE-821 versus BMS-754807/VE-821 combination. N=3, P<0.05 **F.** Calculation of Synergy effects using CompuSyn Software. Where a Combination Index (CI) <1, two drugs are synergistic, where CI = 1, two drugs are additive and where a CI>1 two drugs are said to antagonistic.

Noting the potential therapeutic benefit of dual inhibition of IGF-1R and ATR, we next investigated whether the DDR contributes to resistance to the IGF-1R inhibitor BMS-754807. An MCF-7 cell line that is resistant to BMS-754807 was generated by exposure to increasing concentrations of the inhibitor over time, until cells were cultured in the presence of drug at a 500nM concentration. The IC50 of these resistant cells (designated MCF-7-R) was then compared to parental MCF-7 cells and found to be more than 7 fold higher (Figure 4A). In the presence of 500nM BMS-754807, parental MCF-7 cells induced apoptotic pathways leading to an increase in the levels of cleaved PARP. However in the presence of a similar concentration of inhibitor, MCF-7-R cells did not show cleavage of PARP, indicating that these cells do not undergo apoptosis when exposed to this concentration of BMS-754807 (Figure 4B).

We next asked whether these resistant cells exhibited γH2AX induction and activation of DNA damage pathways. Parental MCF-7 cells were exposed to 500nM BMS-754807 and compared to MCF-7-R cells. Parental MCF-7 cells demonstrated a large increase in γ-H2AX and phosphorylation of Chk1compared to the untreated controls (Figure 4C). Intriguingly, MCF-7-R cells cultured in the presence of BMS-754807 retained induction of γH2AX and phosphorylation of Chk1 similar to that observed in parental cells exposed to the drug (Figure 4C). This indicates that IGF-1R inhibition may still activate the DNA Damage repair pathways in cells made resistant to IGF-1R kinase inhibition, and suggests that MCF-7-R cells may be sensitive to IGF-1R inhibition in the presence of the ATR inhibitor. To test this concept further we examined whether dual inhibition of IGF-1R and ATR could reverse some of the resistant phenotype seen in these cells. Dual exposure to BMS-754807 and VE-821 led to a visible reduction in colony growth compared to either drug alone (Figure 4D), and consistently led to a decrease in colonies across a range of concentrations (Figure 4E). MCF-7-R cells appeared to be clearly more sensitive to co-inhibition of IGF-1R and ATR than parental cells. Drug synergy analysis indicated that the relationship between the two drugs was strongly synergistic within the ranges of 30-100nM BMS-754807 and 300-1000nM VE-821 (Figure 4F). These results collectively indicate that MCF-7 cells resistant to the IGF-1R inhibitor BMS-754807 continue to induce activation of the ATR pathway in response to IGF-1R inhibition, and given the lower CI values, are more sensitive to ATR inhibition than their parental counterparts. Thus, IGF-1R kinase inhibition combined with ATR inhibition may have particular therapeutic benefit in cases of acquired resistance to IGF-1R TKIs.

### IGF-1R inhibition sensitizes breast cancer cells to cisplatin

**Figure 5:**
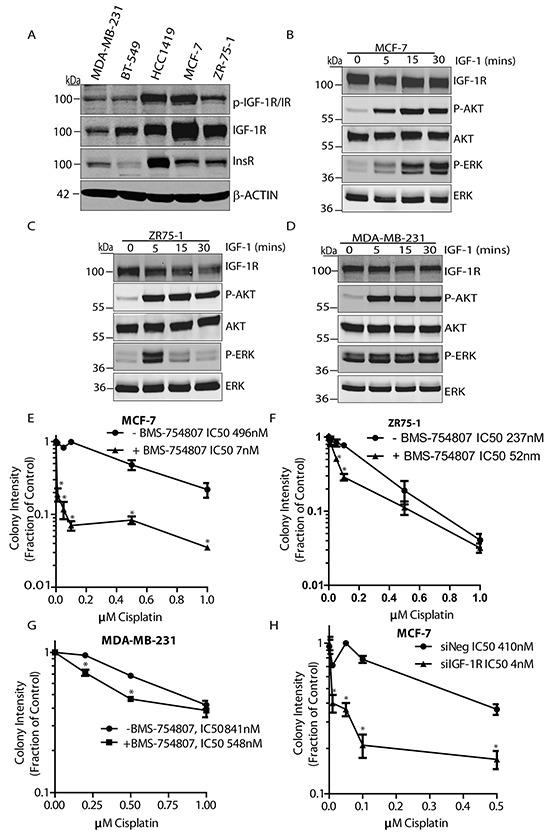
IGF-1R inhibition sensitizes breast cancer cells to cisplatin **A.** Whole cell lysates from MDA-MB-231, BT-549, HCC1419, MCF-7 and ZR-75-1 cell lines were isolated and immunoblotted with anti-phopho-IGF-1R/IR, anti-IGF-1R, anti-IR, or anti-β-Actin antibodies. **B.** MCF-7 cells were serum-deprived for 4 hours, stimulated with IGF-1 and immunoblotted with anti-IGF-1R, anti-phospho-AKT, anti-AKT, anti-phospho-ERK, or anti-ERK antibodies. **C.** ZR-75-1 cells were serum-deprived for 4 hours, stimulated with IGF-1 and immunoblotted with anti-IGF-1R, anti-phospho-AKT, anti-AKT, anti-phospho-ERK, or anti-ERK antibodies. **D.** MDA-MB-231 cells were serum-deprived for 4 hours, stimulated with IGF-1 and immunoblotted with anti-IGF-1R, anti-phospho-AKT, anti-AKT, anti-phospho-ERK, or anti-ERK antibodies. **E.** Survival curve for MCF-7 cells incubated in complete medium with or without 30nM BMS-754807 and increasing concentrations of cisplatin. Cells were seeded into colony formation assays and allowed to grow for 14 days. Odyssey Infrared Imaging system was used to quantify colonies and results are expressed as a fraction of untreated controls (Log_10_ scale). Student-T-Test, n=3, *p<0.05 **F.** Survival curve for ZR75-1 cells incubated with or without 120nM BMS-754807 and increasing concentrations of cisplatin. Cells were seeded into colony formation assays and allowed to grow for 14 days. Odyssey Infrared Imaging system was used to quantify colonies and results are expressed as a fraction of untreated controls (Log_10_ scale). Student-T-Test, n=3, *p<0.05 **G.** Survival curve for MDA-MB-231 cells incubated with or without 300nM BMS-754807 and increasing concentrations of cisplatin. Cells were seeded into colony formation assays and allowed to grow for 14 days. The Odyssey Infrared Imaging system was used to quantify colonies and results are expressed as a fraction of untreated controls (Log_10_ scale). Student-T-Test, n=3, *p<0.05 **H.** Survival curve for cisplatin treatment in MCF-7 cells following IGF-1R siRNA knockdown. Cells were transfected with IGF-1R-targeting siRNA for 24 hrs before being seeded into colony formation assays in the presence of increasing concentrations of cisplatin. The Odyssey Infrared Imaging system was used to quantify colonies and results are expressed as a fraction of untreated controls (Log_10_ scale). Student-T-Test, n=3, *p<0.05.

DNA damaging agents have a critical role in cancer therapy, so we next investigated the effect of combining IGF-1R inhibition and cisplatin in three breast cancer cell lines MCF-7, ZR-75-1 and MDA-MB-231 using colony formation assays. IGF-1R expression levels are different in these cell lines (Figure 5A), with MCF-7 exhibiting the highest IGF-1R expression followed by ZR-75-1 and MDA-MB-231 cells, while all 3 cell lines are responsive to IGF-1 stimulation as determined by phosphorylation of AKT and ERK (Figure 5B-5D).

MCF-7, ZR-75-1, and MDA-MB-231 cells were each exposed to BMS-754807 at 30nM, 120nM, or 300nM in the presence of cisplatin at concentrations ranging from 0 to 1.0 μM. BMS-754807 significantly sensitized all cell lines to cisplatin, reducing the IC50 from 496nM to 4nM in MCF-7 cells (Figure 5E), from 237nM to 52nM in ZR-75-1 cells (Figure 5F), and from 841nM to 584nM in the more resistant MDA-MB-231 cells (Figure 5G). A similar effect was observed following siRNA-mediated suppression of IGF-1R in MCF-7 cells, where the IC50 decreased from 410nM to 7nM (Figure 5H). These results indicate that IGF-1R suppression enhances the potential of cisplatin therapy in phenotypically distinct breast cancer cell lines, and further indicates the benefit of combining IGF-1R inhibitors with DNA damaging agents.

Overall, these results indicate that IGF-1R inhibition may be effective in combination with cisplatin in breast cancer cells that exhibit different levels of sensitivity to either the TKI or cisplatin.

## DISCUSSION

Many cancers have dysfunctional DNA repair systems, enabling bypass of cell cycle arrest and continued cell division with damaged DNA, which then renders these cells more susceptible to extreme DNA damage caused by chemotherapy and radiotherapy [39, 40]. Double or single stranded breaks in DNA normally activate DNA damage repair kinases, ATM and ATR to maintain genome stability. The ATR inhibitor VE-821 has been shown to significantly enhance the sensitivity of pancreatic cancer cells to radiation and gemcitabine [41], to enhance the sensitivity of ovarian cell lines to cisplatin, topotecan, and veliparib [42], and to radiosensitize human promyelocyte leukaemia cells [43]. In general it is considered that by inhibiting this key component of the DNA damage repair pathways, cancer cells are rendered more sensitive to agents that cause DNA damage. Previous work has shown that DNA damage sensed by ATM, a kinase related to ATR, can induce IGF-1 expression to drive pro-survival signals following chemotherapy or radiation [44].

**Figure 6:**
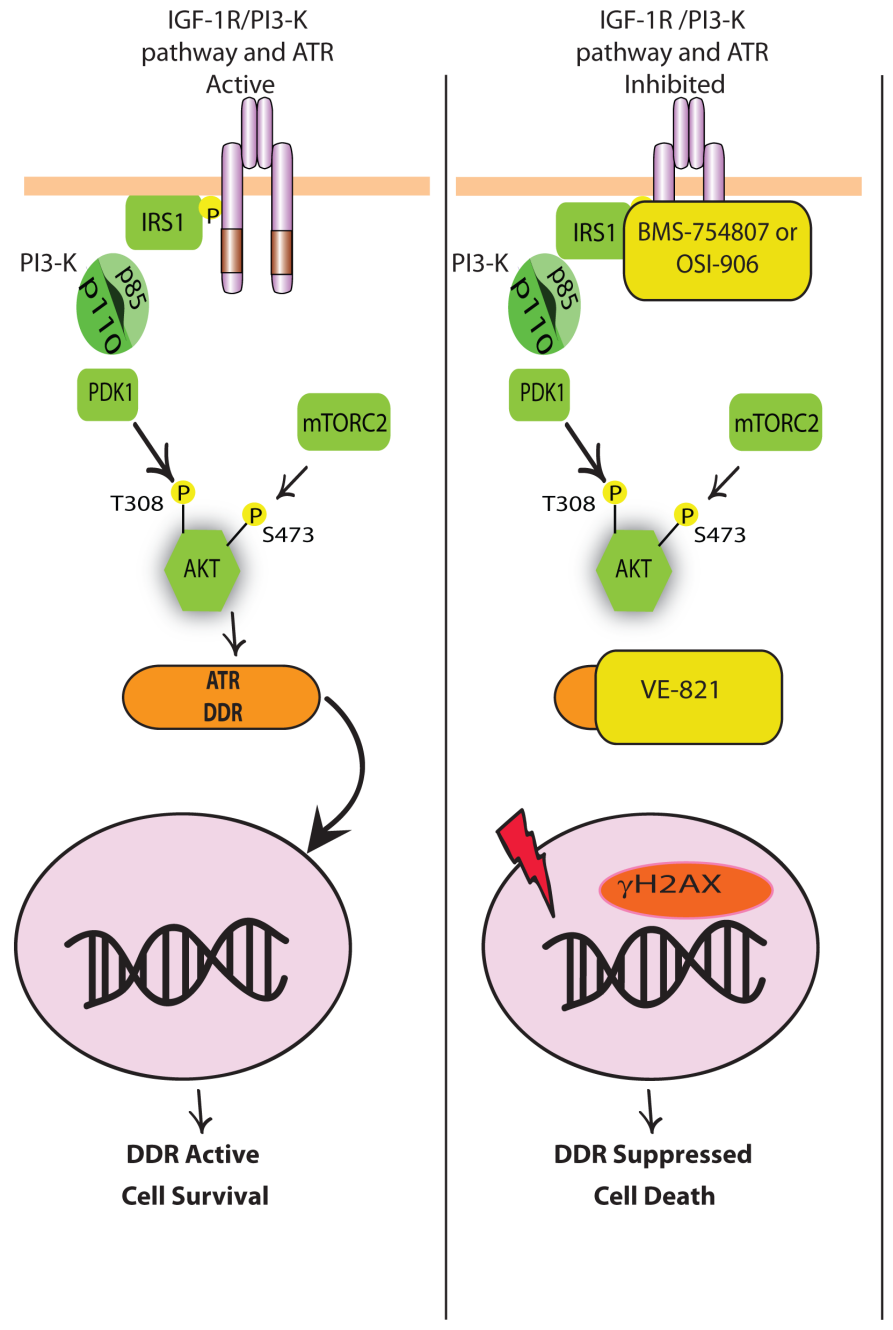
Schematic model for how dual inhibition of IGF-1R and ATR leads to increased cell death (Left) IGF-1 binding to IGF-1R leads to phosphorylation and activation of the IGF-1R and recruitment of IRS-1 for the activation of the PI3-K pathway. Following activation of PI3-K, AKT becomes phosphorylated on two key residues by PDK1 and TORC2. This allows for AKT to engage in pro-survival signalling. When the IGF-1R-PI3-K-AKT pathway is active, cells are protected from the cytotoxic effects of chemotherapies and can activate DDR and anti-apoptotic pathways to prevent cell death. *(Right)* In the presence of IGF-1R TKIs OSI-906 or BMS-754807, activation of the IGF-1R and PI3-K pathway are inhibited and DNA damage is induced in the nucleus (γH2AX). In response to γH2AX, ATR and other components of the DDR response are activated to repair DNA. However in the presence of VE-821, ATR cannot repair the damaged DNA and cell death occurs.

Our results are, to our knowledge, the first to provide evidence for the potential benefit of co-targeting the IGF-1R and ATR, and the results of this study are summarized in Figure 6. We propose that inhibition of IGF-1R induces concurrent activation of the DNA damage sensor ATR, knockdown of which is sufficient to sensitize cells to BMS-754807. Importantly, in MCF-7 cells that had acquired resistance to BMS-754807, the effects of combining IGF-1R and ATR kinase inhibitors resulted in more pronounced synergistic cytotoxicity than that observed in sensitive cells. Interestingly, MCF-7-R cells exhibited stronger synergy between BMS-754807 and VE-821 than MCF-7 parental cells indicating that cells that are resistant to BMS-754807 may be more sensitive to this drug combination. This suggests potential for therapeutic combination of these two agents in cancers with acquired resistance to TKIs in the clinical setting. It may also be possible that initial treatment with the combination may prevent the emergence of IGF-1R inhibitor resistance by selectively targeting ATR-activated clones.

IGF-1R inhibition has been found to delay both non-homologous end-joining and homologous recombination [29]. Therefore exposure to an IGF-1R inhibitor such as BMS-754807 could delay DNA damage repair and therefore ‘prime’ cancer cells for treatment with a DNA damaging agent. This could make the cells more sensitive to inhibition of ATR. Indeed, ATR inhibition preferentially targets HR-deficient cancer cells [45]. Therefore therapies which delay HR would be beneficial in combination with ATR inhibitors. Indeed in prostate cancers cells, suppression of RAD51, the recombinase that catalyses the strand invasion step of HR, sensitises cells to IGF-1R inhibition [35].

TKIs that inhibit the IGF-1R also inhibit the homologous Insulin Receptor kinase, so it is possible that some of the effects are caused by inhibition of IR activity. However, our data herein and previous reports strongly indicate that the effects are largely driven by IGF-1R inhibition because suppression of IGF-1R is sufficient to induce DNA damage [29, 35], and to prevent induction of DNA damage by IGF-1R TKIs. This conclusion is also supported by a study investigating the mechanism of action of BMS-754807 where RNA profiling analysis was used to compare its effects with those of IGF-1R knockout [46]. The results indicated that although BMS-754807 inhibits both IGF-IR and IR, many of the gene expression changes caused by BMS-754807 were due to IGF-IR inhibition alone.

Inhibition of the PI3-K pathway appears to be required for the effects of IGF-1R inhibitors in inducing DNA damage. The AKT-PI3-K pathway has been linked to sensitivity to IGF-1R inhibition whereby cells over-expressing components of the IGF-1R/PI3-K signalling axis were more sensitive to IGF-1R inhibition [47, 48]. This effect may well be may be linked to induction of DNA damage as observed in our study. Our data therefore suggested that combining selective inhibitors of PI3-K and ATR may also have synergistic therapeutic effects. Interestingly, a recent study in TNBC cell lines indicates beneficial effects from combining an IGF-1R/IR inhibitor (OSI-906) with a PI3K inhibitor (GDC-0491), which indicates that PI3-K is activated independently of IGF-1R activity [49].

Either IGF-1R kinase inhibitors or siRNA-mediated suppression of IGF-1R expression is sufficient to sensitize breast cancer cells to cisplatin treatment. Interestingly MCF-7 cells exhibited the greatest increase in sensitivity to cisplatin upon inhibition of the IGF-1R. This cell line has the highest expression of IGF-1R among those tested, and has been previously shown to be sensitive to IGF-1R inhibition [30]. Though not a common therapy for all breast cancers, cisplatin is being investigated for use in triple negative breast cancers, in which IGF-1R has been shown to have high activity [30]. The IGF-1R pathway was observed to be up-regulated in microarray analysis of Ovarian Cancer tissues while also inversely correlating with survival [50]. Moreover, hyper-activation of IGF-1R has been found to be essential for cisplatin resistance in ovarian cancer [51]. This suggests that the IGF-1R may be a potential co-targeting option for other cancers such as ovarian cancer that are currently treated with cisplatin. Despite much research there are currently no reliable biomarkers available to predict response [19] to IGF-1R inhibition. IGF-1R expression levels do not appear to predict IGF-1R activity [52], and the differential expression of signalling components in cancer cells that modulate IGF-1R activity may contribute to sensitivity/resistance to anti-IGF-1R therapies (reviewed in [12]). This modulation has been attributed to differential activation of PI3-K and MAPK pathway components [47, 48, 53, 54] as well as expression of alternative RTKs and Integrin receptors [55–57]. It is likely that cancers that are reliant on IGF-1R signalling will exhibit the greatest benefit from co-targeting, which again highlights the lack of biomarkers for IGF-1R activity as a key challenge.

In summary our study provides a mechanistic rationale for the combination of IGF-1R or IGF pathway inhibitor with ATR inhibitors or other DNA damaging agents in breast cancer.

## MATERIALS AND METHODS

### Materials

Recombinant IGF-1 was from PeproTech (Rocky Hill, NJ). BMS-754807 was purchased from Active Biochem, PD98059 and LY294002 were from Merck, VE-821and OSI-906 were from Selleckchem. Customized siRNA Library was from Qiagen. Antibodies specific for phospho-H2AX (Rabbit, #9718), phospho-Chk1 (Rabbit, #2348s), Phospho-AKT (Rabbit, # 4060s), AKT (Mouse, #2920s), phospho-ERK (Rabbit, #4941), and phospho-IGF1R Y1135/1136 (Rabbit #3024) were from Cell Signalling Technology, Danvers, MA. Anti-Chk1 (Mouse, #sc-8408), Anti-IGF1Rβ (Rabbit, #sc-711) and anti-α-Tubulin (Mouse, #sc-23948) antibodies were from Santa Cruz Biotechnology, Santa Cruz, CA. Anti-β-Actin (Mouse, #A5441) antibody was from Sigma Aldrich. Anti-ERK2 (Mouse, #4696) antibody was from Millipore.

### Cell culture, IGF-1 mediated stimulation of cells and viability assays

MCF-7 and ZR-75-1 cells were maintained in DMEM or RMPI medium, respectively, supplemented with 10% foetal bovine serum and 10mM L-Glutamine and 5 mg/ml penicillin/streptomycin. Unless otherwise stated, western blot analysis of whole cell lysates was conducted from cells maintained in complete media with or without appropriate treatments. For analysis of cell signalling in response to IGF-1, cells were generally cultured at seeding densities that allowed for an approximate 70% confluency after 16 hours. Adhered cells were then washed three times with serum-free media (DMEM with 10mM L-Glutamine) and maintained in serum-free media for a total of 4 hours followed by stimulation with 10ng/ml IGF-1 for 10 minutes.

Cell viability was assessed using Cell Titre Glo (Promega, Madison, WI), which measures ATP. Cells were seeded into 96 well plates, and 24 hrs later were incubated with BMS-754807 for a further 48 hrs. Cells were then lysed using Cell Titre Glo reagent and analysed using a spectrophotometer.

### Oncogene and tumour suppressor gene siRNA library

Three independent siRNAs for each target tumour suppressor or oncogene were arrayed in a 96-well platform. MCF-7 cells were reverse transfected with 10nM siRNA for 24 hrs, after which cells were treated with 30nM BMS or vehicle control for 48 hrs. Viability was assessed using Cell Titre Glo as above. Results were median-centred; log transformed and normalized to scrambled siRNA controls. Hits with robust Z-scores of at least +/− 1MAD from the scrambled siRNA were selected and carried forward for pathway analysis, while hits with at least +/− 3MAD were carried forward for validation studies.

To suppress IGF-1R expression specifically, two individual oligos targeting the IGF-1R were obtained from Qiagen (cat # S100017521 and S102624552). A non-targeting oligonucleotide, Silencer Negative siRNA Control #2 (AM4311) from Ambion (Cambridgeshire, UK) was used as a Negative Control. Transfections were performed with a final concentration of 20nM siRNA using RNAiMAX. For siRNA transfection, cells were trypsinsed and re-suspended in Penicillin/Streptomycin-free culture media. siRNA oligonucleotides were diluted to a final volume of 162.5 μl in OptiMem media. RNAiMAX transfection reagent (7.5 μl in 155 μl of OptiMem) was added to the siRNA solutions and samples were incubated for 5 minutes. 300 μl of the siRNA/RNAiMAX complex was pipetted to 1 well of a 6-well plate and 8.5 × 105 cells (in a final volume of 1.2ml) were then added. Cells were allowed to adhere overnight to the tissue culture plate, in the presence of the siRNA/RNAiMAX complex. 24 hours post-transfection, the siRNA/RNAiMAX complex was removed and cells were reseeded for experiments. Suppression of IGF-1R was detected by western blotting with anti-IGF-1R antibodies.

#### Stable suppression of IGF-1R

For stable suppression of the IGF-1R cells pGIPZ shRNA scramble vector (catalogue ID RHS 4346) along with pGIPZ IGFIR ShRNA-1 (RHS4430-98705133) and pGIPZ IGFIR shRNA-2 (RHS 4430-98485874) were transfected into MCF-7 cells. Briefly, MCF-7 cells were seeded at a density of 1.2x 10^6^ cells/10 cm tissue culture dish, in Penicillin/Streptomycin-free medium. Eighteen hours later, cells were transfected with relevant plasmids. Solution A was prepared containing 10 μg DNA/10 cm tissue culture dish diluted in OptiMem serum-free medium to 400 μl in volume. Solution B was prepared containing Lipofectamine 2000 at 20 μl per 10 cm tissue culture dish diluted in OptiMem to 400 μl in volume. Both Solution A and B were incubated at room temperature for 5 minutes in the dark prior to mixing at a ratio of 1:1 and further incubation at room temperature for 20 minutes. The cells to be transfected were washed twice with OptiMem, prior to addition of 800μl of the DNA/Lipofectamine in a drop-wise fashion. 2.2ml of additional OptiMem was then added to each plate. Following this, the transfected cells were incubated at 37°C overnight. Selection of stable pools was then immediately commenced in DMEM complete media containing puromycin at a concentration of 0.5μg/ml. Drug containing media was regularly replenished and selection was deemed complete when all cells in the untransfected control plate where dead (~6days).

### Colony formation assays

Drug sensitivity was assessed using colony formation assays. 2×10^3^ (MCF-7 cell) and 4×10^3^ (MCF-7-R cells) were seeded into each well of a 6 well plate. 24 hrs later, cells were incubated with varying concentrations of drug to determine a concentration that was cytotoxic to approximately 50% of the cells. For cisplatin and BMS-754807 combinations, cells were treated with a concentration of drug that killed approximately 30% of the cells. Cells were transfected with siRNA for 24 hours prior to seeding in tissue culture plates at low density and were allowed to grow for up to 21 days, or until colony growth was visible in control wells. Colonies were fixed in 95% Ethanol for 10 minutes and stained with 0.05% crystal violet in 0.1% Ethanol for 30 minutes. Colonies present were measured by infrared scanning using Odyssey Scanning Equipment. IC50 values were determined as the concentration that results in a 50% reduction in cell survival. For combination treatments with cisplatin, samples were compared to BMS-754807 treatment alone (100%). All graphs and IC50 calculations were generated using GraphPad Prism software, and results are generally represented on graphs in Log scale as a fraction of the untreated controls.

### Preparation of cellular extracts and western blotting

Cell lysates were generated using RIPA lysis buffer (50mM Tris pH 7.4, 150mM NaCl, 1% NP40, 0.5% Sodium deoxycholate, 0.1% Sodium Dodecyl Sulfate). Immediately prior to cell lysis fresh phosphatase inhibitors, Sodium Orthovanadate (Na3VO4) (1mM), β-Glycerophosphate (βGP) (1mM), and Sodium Pyrophosphate (SPP) (2.5mM), and protease inhibitors (Halt cocktail 1/100 dilution; AEBSF, aprotinin, bestatin, E-64, leupeptin and pepstatin A) were added to lysis buffer. Following incubation on ice for 20 minutes, nuclear and cellular debris were removed by centrifugation at 14000 RPM for 15 minutes at 4°C. Protein concentration was determined via Bradford Assay utilising Bradford protein quantification reagent at a wavelength of 595nm (Spectramax 384). Following estimation of protein concentration, samples were denatured by boiling for 5 minutes in 5X loading buffer (2% w/v SDS, 8% v/v glycerol, 60mM Tris-HCL, pH6.8, 1.2% β-mercaptoethanol, and 0.2-0.4% Bromophenol Blue) and equal amounts of protein were resolved by SDS-PAGE and analysed by western immunoblotting. Nitrocellulose membranes were then blocked for 1 hour at room temperature in Tris-buffered saline containing 0.05% Tween 20 (TBS-T) and 5% Bovine Serum Albumin (w/v). All primary antibody incubations were performed overnight at 4°C, and secondary antibody incubations were for 1 hour at room temperature. Alexa Fluor 680- and 800-coupled anti-rabbit and anti-mouse secondary antibodies (LI-COR Biosciences Cambridge, UK) were used for detection with the Odyssey infrared imaging system (LI-COR Biosciences, Cambridge, UK).

### Generation of BMS-754807 resistant MCF-7 cells

Parental MCF-7 cells were exposed to increasing step-wise increments of BMS-754807 until cells were proliferating successfully in the presence of each increased drug concentration, and a resistant pool had been generated. Cells were then permanently maintained in cell culture medium containing 500nM BMS-754807.

### Statistical and drug relationship analysis

P values were calculated using Student-T-Test, or where specified using 2way-ANOVA (P<0.05). Quantification of drug synergism and antagonism were conducted using CompuSyn Software.

## SUPPLEMENTARY MATERIALS FIGURES


