# Supplementary material for: IGF-1R inhibition sensitizes breast cancer cells to ATM-related kinase (ATR) inhibitor and cisplatin
Source: Oncotarget. 2016 Jul 27;7(35):56826–41. doi: 10.18632/oncotarget.10862 (PMC5302955; doi:10.18632/oncotarget.10862)
Supplement: Supplementary file 1 [file oncotarget-07-56826-s001.pdf]

IGF-1R inhibition sensitizes breast cancer cells to ATM-Related Kinase (ATR) inhibitor and cisplatin

SUPPLEMENTARY FIGURES

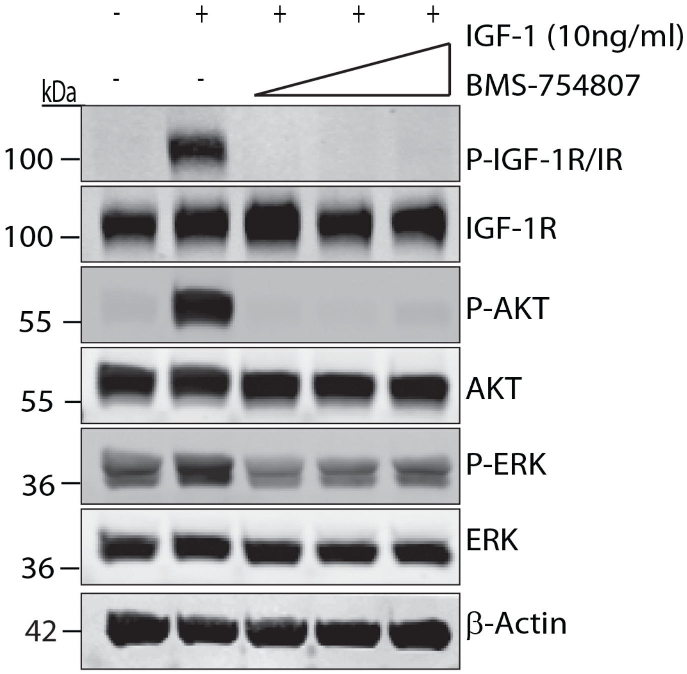

Supplementary Figure S1: BMS-754807 inhibits IGF-1 induced activation of IGF-1R and activation of PI3-K and MAPK pathways. MCF-7 cell cultures were serum-starved for 4 hours and incubated with 100nM, 500nM and 1000nM of BMS-754807 for the final hour of starvation. Cells were then stimulated with 10ng/ml of IGF-1 for 10 minutes, followed by cell lysis and immunoblotting with anti-phopho-IGF-1R/IR, anti-IGF-1R, anti-phospho-AKT, anti-AKT, anti-phospho-ERK, anti-ERK, or anti-β-Actin antibodies.

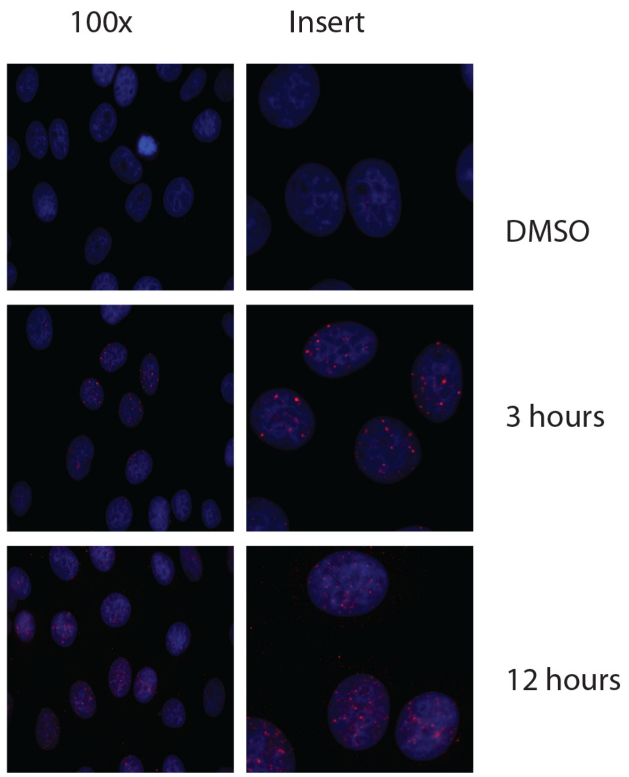

Supplementary Figure S2: BMS-754807 treatment induces γH2AX positive Foci in the nuclei. MCF-7 cells were treated with 30nM of BMS-754807 over a time course followed by fixation and staining with phospho-H2AX antibodies (γH2AX) (red) and Hoechst (Blue).

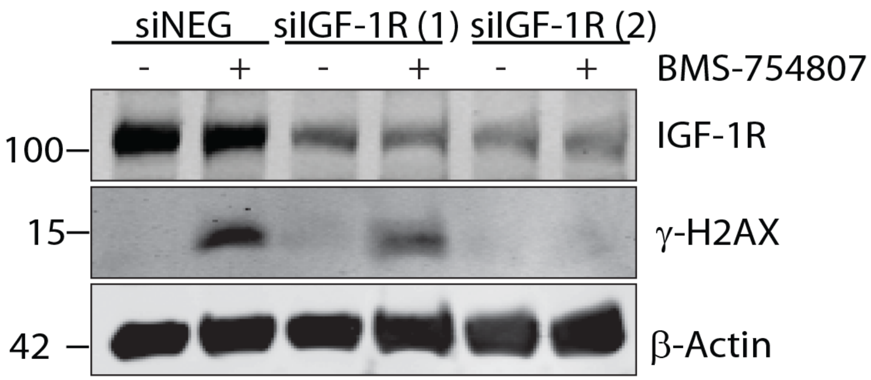

Supplementary Figure S3: SiRNA-mediated suppression of IGF-1R prevents the induction of γH2AX by BMS-754807. MCF-7 cells were transfected with 20nM IGF-1R-targeting siRNAs or a control siRNA, and 48 hrs later were treated with 500 nM BMS-754807 for a further 24 hrs, followed by cell lysis and immunoblotting with anti-IGF-1R, phospho-H2AX (γH2AX) and anti-β-Actin antibodies.

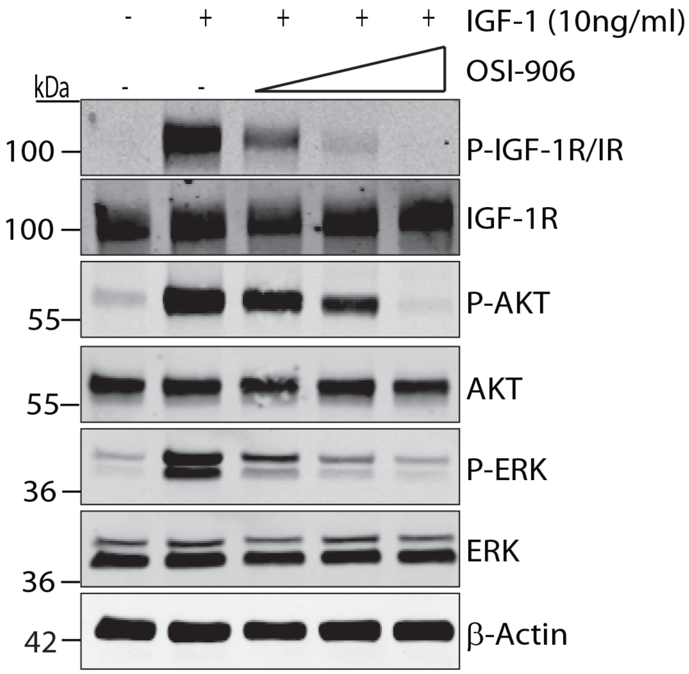

Supplementary Figure S4: OSI-906 inhibits IGF-1 induced activation of IGF-1R and activation of PI3-K and MAPK pathways. MCF-7 cells were serum-starved for 4 hours and incubated with 10nM, 100nM and 1000nM of OSI-906 for the final hour of starvation. Cells were then stimulated with 10ng/ml IGF-1 for 10 minutes, followed by cell lysis and immunoblotting with anti-phopho-IGF-1R/IR, anti-IGF-1R, anti-phospho-AKT, anti-AKT, anti-phospho-ERK, anti-ERK and anti-β-Actin antibodies.

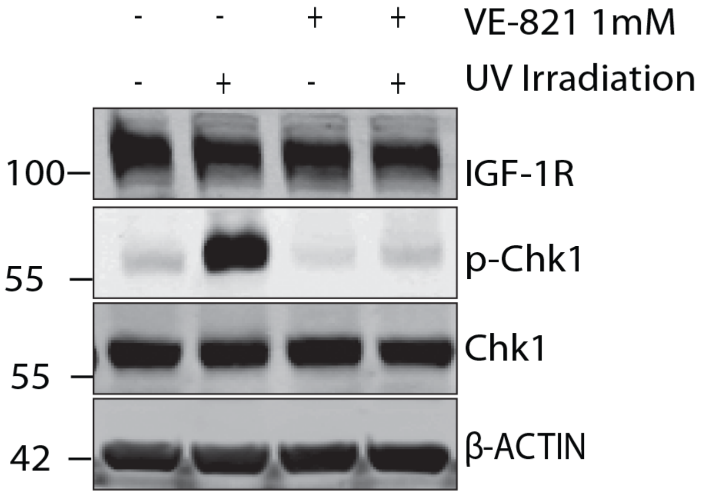

Supplementary Figure S5: VE-821 inhibits ATR activation. MCF-7 cells were pre-treated with 1μM VE-821 for 1 hour followed by exposure to 100J/m2 of ultra violet rays and lysis of cells 2 hours later. Lysates were then resolved by SDS-PAGE and immunoblotted with anti-IGF-1R, anti-phospho-Chk1, anti-Chk1, or anti-β-Actin antibodies.
